# Erosive Tooth Wear and Gastroesophageal Reflux Disease: An Interdisciplinary Management for a Relevant Dental Problem

**DOI:** 10.7759/cureus.84668

**Published:** 2025-05-23

**Authors:** Kim Attanasi, Ashok Hospattankar

**Affiliations:** 1 Medical and Scientific Affairs, Haleon US, New Jersey, USA

**Keywords:** acid reflux, dental erosion, erosive tooth wear, gastroesophageal reflux disease, teledentistry

## Abstract

Gastroesophageal reflux disease (GERD) is a frequently occurring digestive disorder resulting in acid reflux into the oral cavity. Apart from its esophageal symptoms, it also manifests in the mouth, with erosive tooth wear (ETW) being the most encountered oral affliction. ETW is a cumulative and irreversible condition; if it remains unrecognized, it progressively worsens over time, significantly impacting the quality of life and imposing a substantial economic burden, especially when associated with untreated GERD. Early identification and timely intervention are essential to preventing disease progression and minimizing long-term consequences. An interdisciplinary approach that integrates medical and dental care offers the most effective management strategy. Preventive measures, including lifestyle and dietary modifications, patient education on appropriate oral hygiene, and the application of fluoride, play an important role in mitigating ETW risk. This paper explores the pathophysiological link between ETW and untreated GERD, focusing on its etiology, clinical presentation, and diagnostic considerations. It also examines the economic burden of untreated ETW and discusses the potential of emerging technologies, like artificial intelligence, digital and teledentistry in improving early detection and tailored treatment. Additionally, the review outlines effective management strategies aimed at improving clinical outcomes and ensuring comprehensive patient care.

## Introduction and background

Gastroesophageal reflux disease (GERD) is a common chronic digestive disorder characterized by retrograde passage of gastric contents into the oral cavity via the esophagus. This condition causes bothersome symptoms, complications, and a progressive decline in quality of life [1]. Globally, GERD affects approximately 1.03 billion people, accounting for 13.98% of the population [2]. In the United States (US), its prevalence ranges between 18.1% and 27.8% [3]. Studies identify age as a key risk factor for GERD, with a significantly higher incidence observed among young adults aged 30 and 39 years [2,4]. The impact of GERD extends beyond typical esophageal symptoms, such as heartburn and acid regurgitation. It also manifests in the oral cavity [5]. If left untreated, GERD can lead to serious consequences, such as esophagitis and Barrett's esophagus [3].

Erosive tooth wear (ETW), a common oral manifestation of GERD, involves the chemical loss of the mineralized tooth structure due to acid exposure unrelated to oral bacteria [6-8]. ETW is progressive, irreversible, and cumulative, with its effects worsening over time, especially when it begins in childhood [9]. As individuals age, ETW can lead to complications such as dentinal hypersensitivity, yellowish tooth discoloration, loss of occlusal balance, difficulties with dental restorations, functional impairments, and increased dental costs [5,8,10]. Although ETW is irreversible, its progression can be halted with appropriate management [9]. Therefore, early diagnosis and intervention are crucial to prevent long-term dental complications and the need for complex restorative therapy. However, in the initial stage, ETW may exhibit subtle signs that are challenging to identify during routine examinations [5].

Numerous studies have shown an association between GERD and ETW [11]. Patients with GERD often experience severe erosive disease due to persistent acid reflux, which accelerates the dissolution of dental hard tissue [11]. The prevalence of ETW in GERD patients shows wide variation, ranging between 25% and 80% [11]. Considering the growing dental concerns related to ETW caused by untreated GERD, which can significantly impact oral health, it is crucial to identify, prevent, and manage ETW early. Dental health care professionals must stay informed about this condition and possess comprehensive knowledge of its implications.

This paper aims to provide current understanding of ETW associated with untreated GERD, including its etiology, symptoms, early clinical diagnosis, and economic burden. It explores the challenges associated with currently available diagnostic methods and discusses the role of new technologies like artificial intelligence, digital, and teledentistry in early identification of ETW in GERD patients. The paper also outlines preventive and management strategies with a highlight on the importance of a collaborative or interdisciplinary approach that involves gastroenterologists, dentists, nutritionists, etc. This comprehensive understanding aims to equip dental and healthcare professionals (HCPs) with the knowledge to guide GERD patients in making informed lifestyle choices and managing ETW effectively.

## Review

Methodology

In this narrative review, an electronic search was performed on PubMed, Google Scholar, and ProQuest without any time restrictions using combinations of the following basic and Medical Subject Headings terms: "gastroesophageal reflux disease”, "GERD”, “acid reflux”, “reflux disease” and “dental erosion", “erosive tooth wear”, “tooth wear", erosion, “ETW”. The search and selection process of articles is depicted in the Appendix. The inclusion criteria for the review encompassed studies focusing on adult patients diagnosed with GERD and experiencing ETW, providing insights into the pathophysiological link between GERD and ETW, including etiology, clinical presentation, and diagnostic considerations. Studies exploring the economic burden of untreated ETW and discussing emerging technologies like artificial intelligence (AI) and teledentistry in improving early detection and tailored treatment were included. Additionally, studies outlining effective management strategies aimed at improving clinical outcomes and ensuring comprehensive patient care were considered. The exclusion criteria eliminated studies involving patients with previous upper digestive tract surgery, contraindications for diagnostic procedures, recent use of GERD medications, pregnancy, confirmed or suspected malignancy, and mental health impairments.

Epidemiology of ETW in GERD patients

**Figure 1:**
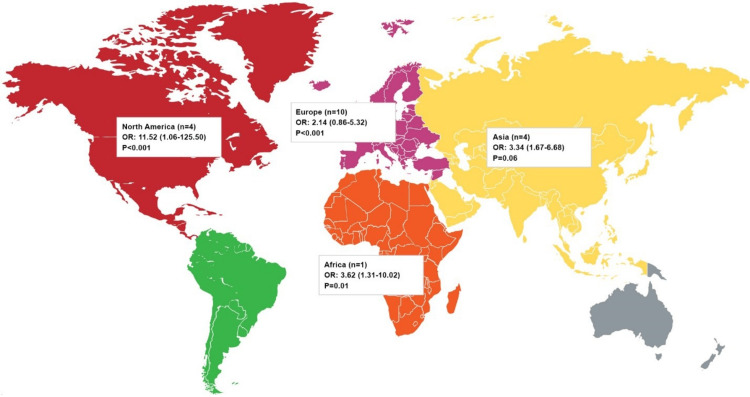
Pooled frequency of ETW in patients with GERD in different regions of the world Adapted and recreated from reference [15] ETW: Erosive tooth wear; GERD: Gastroesophageal reflux disease; OR: Odds ratio; n: number of studies.

The literature shows that the prevalence of ETW, in general, is increasing globally and varies markedly across countries. Several studies report that the mean prevalence of ETW in permanent teeth ranges from 20% to 45% and in deciduous teeth, it ranges from 30% to 50% [12]. In the US, although the data on ETW prevalence is sparse, available studies show a prevalence of 46% among children and up to 80% among adults [13,14]. Epidemiological studies have shown that GERD constitutes an important risk factor for ETW with a positive association between the two conditions [11]. Figure 1 depicts the pooled frequency of ETW in patients with GERD across different geographies [15].

Patients with GERD have a 2-4-fold increased odds ratio of presenting with ETW compared with patients without GERD [15]. A systematic review reported that the mean prevalence of dental erosion in children with GERD was higher than in healthy children (57.2%; SD:34.5 vs. 12.5%; SD:9.3) [16]. The studies included were heterogeneous in diagnostic methodology but they suggested the probable involvement of gastric acid reflux in ETW development. In adult patients with GERD, the prevalence of ETW ranged between 14.4% and 98.1%, with a mean prevalence of 83%, reflecting a strong association between both conditions in adults [17,18]. Furthermore, a high association between GERD and ETW was also found in special populations like the neurodivergent community, 46% had ETW, of which 65% had GERD [11,19]. Likewise, the adult psychiatric inpatients showed a strong association between ETW and gastric reflux (adjusted OR=2.1; 95%CI=1.3-6.2, P<0.001) [11,20].

In children with special needs, the frequency of ETW was significantly higher in those with GERD compared to children without gastric reflux (86.6% vs. 33.7%, P = 0.0001) [11]. Patients with a longer duration of pH<4 had a higher risk of developing ETW [19].

Etiopathogenesis of ETW in GERD patients

**Figure 2:**
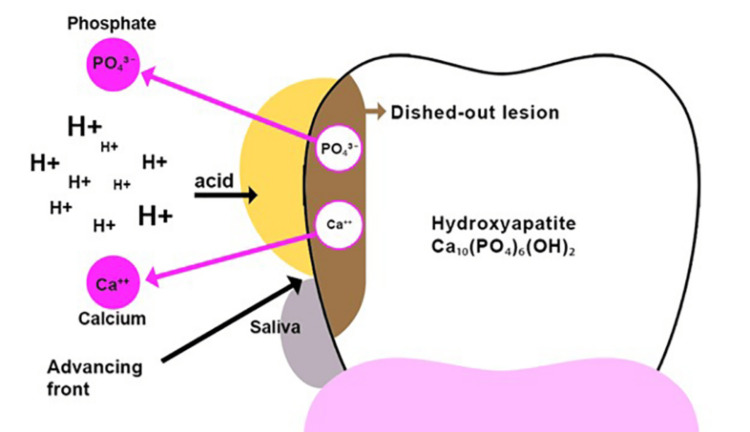
Pathophysiology of ETW in patients with GERD Adapted and recreated from references [5,8,21] Salivary pellicle is removed by repeated acid attacks, which allows the acid to come into direct contact with the tooth surface. Since acid has a lower surface tension than saliva, it easily displaces the saliva and contacts the tooth surfaces. This creates an open system where the tooth surface undergoes demineralization as tooth substance is lost into the oral environment. On the enamel surface, the acid, along with its hydrogen ion (or a chelating agent), starts to dissolve the enamel crystal. This process begins in the prism sheath area and then affects the prism core and the interprismatic areas, leading to the outflow of mineral ions from tooth surface. Consequently, there is a rise in local pH within the tooth substance and in the liquid layer near the enamel surface. This results in a honeycomb appearance or dished-out lesion on the enamel surface. The erosive process is halted in the absence of further acid exposure. ETW: Erosive tooth wear; GERD: Gastroesophageal reflux disease.

The etiopathogenesis of ETW in GERD patients involves a complex pathophysiology resulting in acid dissolution and demineralization of tooth hard tissues (Figure 2) [8,16]. The etiology is multifactorial, involving the direct erosive effects of gastric acid, compromised salivary defense mechanisms, detrimental oral hygiene practices, dietary and lifestyle factors, and underlying medical conditions [5,8,21].

Composition, Frequency, and Duration of Acid Reflux

Acid refluxate may contain partially digested food particles, pepsin, bile acids, or even trypsin (a pancreatic enzyme) in case of duodenal regurgitation [5,8,21]. The pH of gastric acid is approximately 1.2; however, the pH of refluxate can vary depending on its composition [5,8]. The potential and severity of ETW in patients with GERD are influenced by several factors, including the composition and pH of the refluxate, the frequency and form in which it reaches the mouth (whether through regurgitation or belching of acidic vapors), and the number and duration of reflux episodes. Furthermore, the timing of reflux (daytime vs. nocturnal reflux), the duration of GERD, and how far the refluxate travels up the esophagus also play critical roles in determining ETW severity [5,8,21].

Salivary Factors

Some GERD patients may experience reduced salivary flow, which diminishes the natural protective effects of saliva, such as buffering esophageal acid, or clearance of acid reflux [5,8]. A significant association between GERD, hyposalivation, and xerostomia has been observed [22,23]. Reduced salivary flow or hyposalivation predisposes the individual to increased risk for ETW [5,8,21]. Even when saliva is present, its ability to neutralize acid may be overwhelmed in GERD patients, leading to prolonged acidic conditions in the mouth [5,8]. Loss of acquired salivary pellicle due to acids further results in a conducive environment for acid erosion [5,8,21]. The critical pH for demineralization of enamel is approximately 5.5 (and even higher for dentin), which may readily be exceeded by the regurgitated gastric contents. Thus, the likelihood of ETW depends largely on salivary pH [5,10,21].

Behavioral and Lifestyle Factors

Behavioral and lifestyle factors play a significant role in the frequency and severity of acid reflux episodes. Eating patterns such as consuming large meals, excessive intake of beverages, chocolate, caffeine, peppermint, spicy and fatty foods, or lying down soon after eating can trigger more frequent reflux episodes [24]. Additionally, alcohol and tobacco use can relax the lower esophageal sphincter, increasing the likelihood of acid reflux [24]. Tooth brushing habits also contribute to enamel wear; aggressive brushing or brushing teeth immediately after an acid reflux episode can cause more enamel wear due to the softened state of the enamel [5,8]. These factors collectively influence the risk and management of GERD and its associated oral health issues.

Parafunctional Habits

Habits like bruxism, clenching, or grinding can influence ETW. Bruxism is the contact of teeth for reasons other than eating [10]. It can occur during the awake state as tooth clenching, and during sleep as tooth grinding or clenching [10]. Studies show an association between GERD, sleep bruxism and tooth wear, suggesting a synergistic effect that compromises the integrity of dental hard tissues by both chemical (reflux) and mechanical (bruxism) mechanisms [25].

Co-morbid Conditions

Conditions like obesity, diabetes, and respiratory disorders can exacerbate GERD [21,24,26]. Quantifying the prevalence of silent (asymptomatic) GERD is difficult, but population screening studies have estimated the range of 7-10%, with higher rates in people with obesity [27,28].

Medications

Certain medications, such as calcium channel blockers, anticholinergics, and bisphosphonates, can increase the risk of reflux by relaxing the lower esophageal sphincter. Other medicines like aspirin, methamphetamine, etc., are acidic in nature and may cause ETW by directly acting on the teeth [21].

Psychological Factors

Stress, anxiety, and depression can exacerbate GERD symptoms, leading to more frequent acid exposure and subsequent dental erosion [29].

Clinical manifestation and diagnostic techniques

The systemic condition of unrecognized GERD may be evidenced from its oral manifestation, i.e., ETW. Dental professionals therefore can be the first HCPs to diagnose GERD and other related health conditions associated with ETW [21,30]. Notably, a certain degree of physiological tooth wear occurs with ageing [31]. Contrastingly, the pathological condition of ETW lesions that progress relatively faster needs to be identified at an early stage for effective management and prevention of further progression [31].

Assessment of GERD and other associated pre-disposing factors

**Figure 3:**
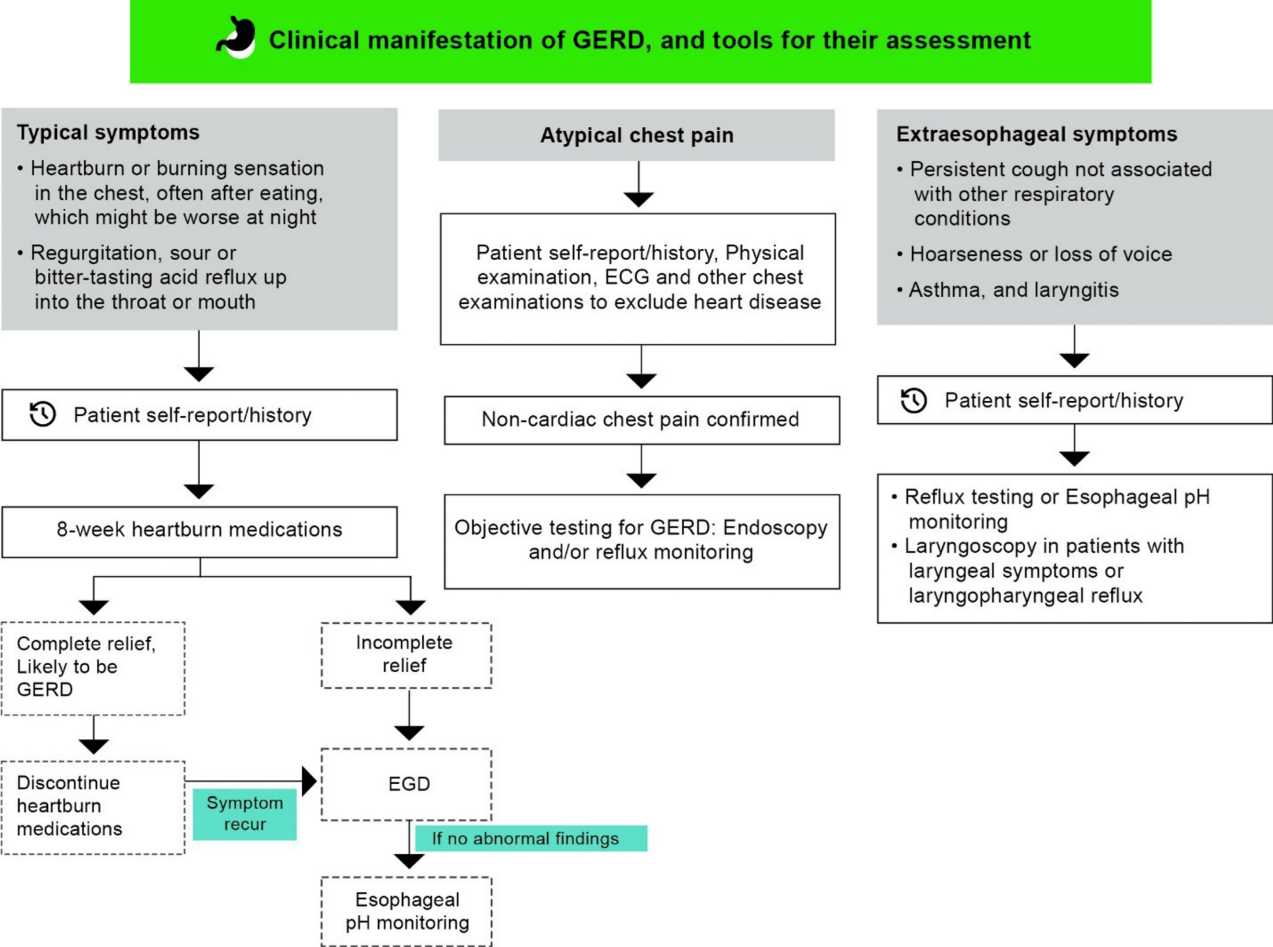
Clinical manifestations of GERD and tools for their assessment Adapted and recreated from references [32-35] ETW: Erosive tooth wear; ECG: Electrocardiogram; EGD: Esophagogastroduodenoscopy; GERD: Gastroesophageal reflux disease.

Appropriate history-taking and clinical assessment are the initial steps to identify GERD. It is usually suspected or diagnosed based on the clinical symptoms and a positive response to heartburn medications [32]. GERD patients commonly experience the classic symptom of heartburn/ acidity along with other symptoms at varying frequencies (Figure 3) [33]. Based on the frequency of symptoms, the disease is classified into three stages: stage I (≤3 episodes per week), stage II (>3 times per week), and stage III (daily symptoms). Postprandial symptom worsening is frequently observed [32]. Symptoms worsen even when lying down or in recumbent positions, particularly during nighttime [33]. In certain patients with persistent GERD symptoms despite high-dose acid suppressant therapies, additional diagnostic assessment is considered [32].

GERD may sometimes be asymptomatic or silent, characterized by mucosal damage without noticeable symptoms. Due to the lack of symptoms, individuals may not seek treatment until more serious issues develop. Also, children with GERD often do not report heartburn until late childhood or early adolescence, causing the condition to remain unrecognized [34,35]. Instead, they may exhibit symptoms such as nausea, abdominal pain, refusal to eat, irritability, poor growth, and unusual weight loss. Dental signs of silent reflux can creep up on patients, and they may not be aware that stomach acid is damaging their teeth until the tooth wear becomes noticeable [5,35]. Patients with GERD experience reduced salivary flow, bruxism, dental caries, dry mouth, burning sensation in the oral mucosa, dysgeusia, halitosis, erythema of the palatal mucosa and uvula [35].

Comprehensive Clinical Examination for ETW

**Figure 4:**
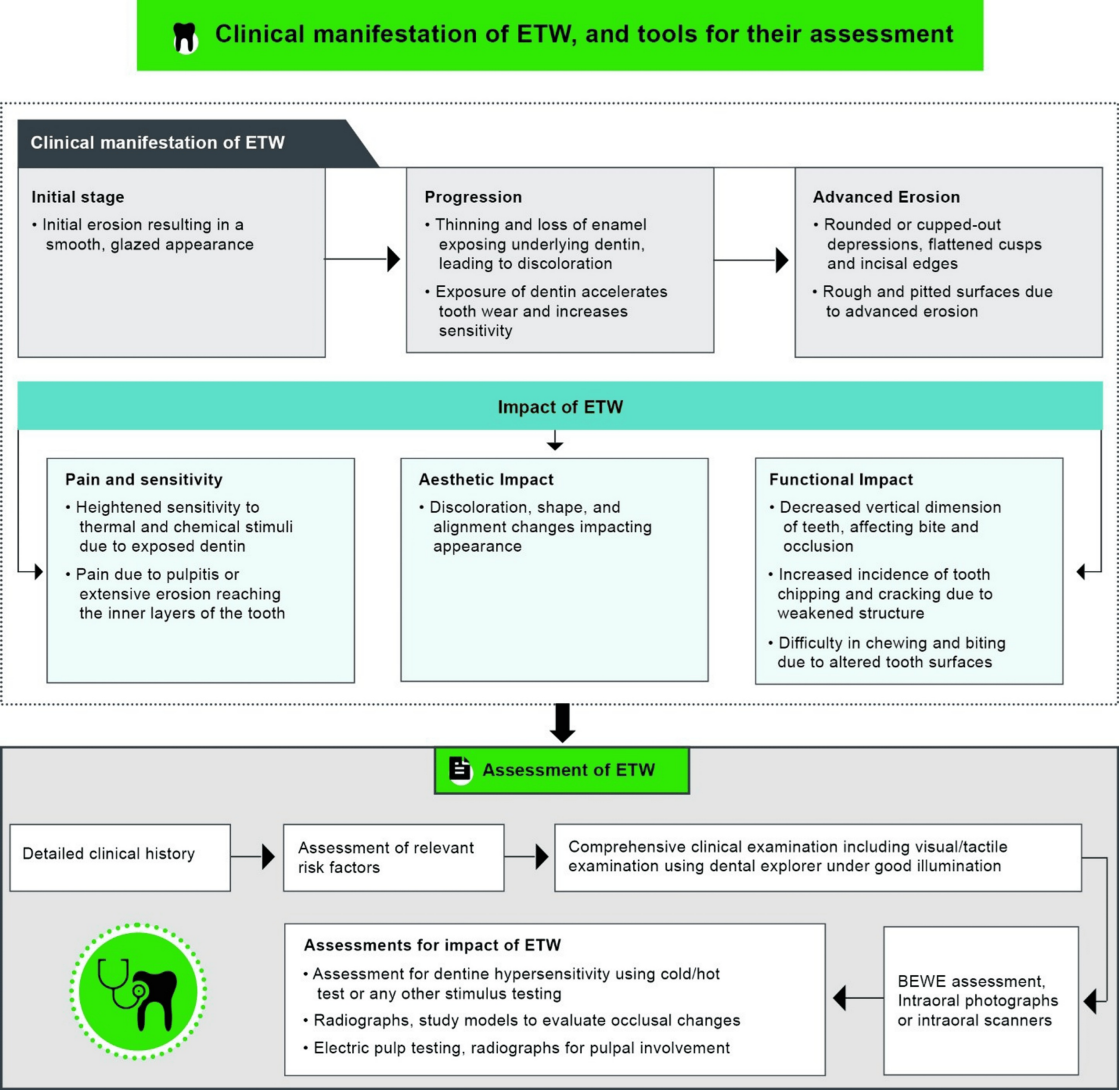
Clinical manifestations of ETW and tools for their assessment Adapted and recreated from references [5,9,35,36] ETW: Erosive tooth wear, BEWE: Basic Erosive Wear Examination

Figure 4 summarizes the clinical manifestations of ETW and their implications [9]. ETW-affected enamel appears thin, smoothly glazed with rounded surfaces, increased incisal and proximal translucency, and yellowish appearance. Tooth wear from grinding or mastication typically occurs more occlusally, while toothbrush abrasion is seen cervically. Other than GERD, ETW may be caused by the intrinsic acids due to eating disorders like anorexia nervosa, bulimia nervosa, and eating disorders not otherwise specified; or extrinsic acids from acidic diets/beverages, or medicinal, occupational, and recreational sources. ETW caused by extrinsic acids shows a generalized pattern of tooth substance loss, whereas intrinsic ETW (due to bulimia or GERD, etc.) primarily affects the palatal surfaces of maxillary incisors and the occlusal surfaces of the mandibular molars in its early stages [9]. Furthermore, the primary dentition is more susceptible to ETW than permanent teeth due to thinner, less mineralized enamel. In 42% of children with GERD-related ETW, the palatal surfaces of posterior teeth are affected [35,36]. Additionally, the absence of the perikymata pattern in maxillary anterior teeth is observed in children [9]. As ETW progresses, the palatal surfaces of the maxillary premolars and molars become affected. In long-standing cases, the occlusal and other surfaces of maxillary and mandibular teeth are also affected, and eventually, the pattern becomes more widespread [35]. Non-eroded restorations often appear raised above the adjacent eroded tooth surfaces [5,9].

Differentiating ETW from Dental Caries

ETW is differentiated from dental caries by the site and type of lesion. Dental caries is confined to areas of biofilm accumulation, affecting both enamel and dentin, primarily the subsurface. In contrast, ETW occurs on plaque-free tooth surfaces exposed to acids. ETW is a surface phenomenon characterized by layer-by-layer dissolution of the dental tissue, giving the tooth a characteristic etched appearance [9].

Diagnostic and monitoring tools for ETW

Study Models/Dental Stone Casts

The progression of ETW can be monitored by comparing the study models from initial and subsequent dental visits. Typically, tooth wear ranges between 11 and 67 µm over a six-month period [30]. However, in cases of untreated GERD, tooth wear progresses significantly faster, with patients experiencing higher tooth surface loss compared to normal individuals (0.18 ± 0.12 mm³ vs. 0.06 ± 0.03 mm³ over six months) [36]. This method, though effective, requires high accuracy in the impression and casting process. Moreover, it takes approximately 3-5 years to identify an accelerated rate of progression, which limits its practical utility [30].

Clinical Indices

There are numerous different clinical indicators/indices available in the literature to diagnose and monitor tooth wear, including ETW. Some frequently discussed indices are summarized in Table 1 [12,37,38]. Clinical indices, whether quantitative or qualitative, help to identify increasing severity or progression of erosion and are usually expressed numerically [30]. These indices enable visualization of surface changes like texture, translucency, and color [30]. However, none of the indices currently have universal acceptance for routine clinical practice [39]. An ideal index for screening ETW in everyday practice should be simple to use, clear in its scoring criteria, and must be reproducible for consistent record-keeping [37]. It is essential to incorporate the selected index into the clinical plan since it assists with ETW diagnosis or monitoring and creates some standardization in the way erosion progression is recorded in clinical notes [40].

**Table 1:** Clinical indices or assessment tools for erosive tooth wear ETW: Erosive tooth wear

Scale/ Index	Specification	Population
Basic Erosive Wear Examination [12,37]	Records the most severely affected surface in each sextant	Children, adults
Eccles Index [12,37]	Severity and site of ETW are recorded	Adults
Exact tooth wear index [12,37]	Comprehensive information on tooth wear is recorded	Adults
Evaluating the index of dental erosion [12,37]	Occurrence of erosive defects and dentine involvement is recorded	Children, adults
Lussi Index [12,37]	Comprehensive information on tooth wear is recorded according to site, dentine exposure and dimension of exposure	Children, adults
Smith and Knight Tooth Wear Index (TWI)[12,37]	Comprehensive information on all four visible surfaces (buccal, cervical, lingual, and occlusal–incisal) of all teeth present are scored for wear	Adults
TWI modified [12,37]	Observation of tooth wear severity	Children
TWI simplified [12,37]	Dentine visibility and extent is recorded on selected teeth/ surfaces	Children, adults
Visual erosion dental examination [12,37]	Loss of enamel or dentine due to ETW is recorded	Adults
Keels-Coffield scale [38]	Severity of ETW is recorded	Children

The International Association of Pediatric Dentistry (IAPD) recommends recording the location and extent of ETW in children using scales like Basic Erosive Wear Examination (BEWE) or Keels-Coffield scale [38]. Amongst all the available indices, the BEWE and the Tooth Wear Evaluation System are the most frequently adopted indices for ETW assessment [30]. The Royal College of England's Clinical Guidelines for Dental Erosion (2021) advocate the BEWE, which is widely recognized as a simple, quick, cost-effective, reliable, and validated tool for use in primary dental care settings [40]. Despite their benefits, clinical indices have limitations. They are subject to clinician interpretation and require a period of approximately one year to 18 months for observable wear changes to manifest [30].

Clinical Photographs

High-quality intraoral and extraoral photographs are valuable for documenting the ETW condition and facilitating comparisons between the initial presentation and follow-up within a diagnostic window of 18 months [30]. Although these photographs cannot capture the depth or texture of the lesions, they provide a useful tool for discussing the condition with patients [30].

Intraoral Scanners and Digital Dentistry in ETW

Digital dentistry refers to the use of advanced technical tools, including specialized software and hardware, to enhance the delivery of dental care. Among these tools, 3D digital intraoral scanners stand out as a potential solution to overcome the subjectivity inherent in clinical assessments. Intraoral scanners are clinical tools that utilize optical sensors to collect data that is processed by software, capturing the 3D morphology and color of tooth surfaces and surrounding structures. These scans enable clinicians to assess the depth, texture, and, to some extent, the color and translucency of erosive lesions. These scans enable clinicians to assess the depth, texture, and, to some extent, color and translucency of erosive lesions [30].

Research suggests that it may take up to two years to assess the ETW progression using scanners. However, these devices offer the advantage of providing quantitative measurement [30]. Newer intraoral scanning software has shown potential for detecting active wear within six months. Despite this progress, challenges related to scan registration, accuracy, and measurement consistency continue to limit their diagnostic potential effectiveness. Advancements in commercially available tooth wear analysis software may help in more effective and quantitative monitoring of ETW in the future. Active screening and clinical monitoring for ETW should be routinely performed in dental practices [30]. Currently, clinical examination and indices remain the mainstay for detection and monitoring of ETW [30]. Clinical photographs are also an essential part of the diagnostic process. Existing methods, including clinical examination and clinical and intraoral photographs, dental casts, or intraoral scans, aid in identifying and monitoring ETW progression [30]. However, their implementation in a clinical setting faces practical challenges [41]. Clinical examinations and indices are subjective, dental casts are labor-intensive, and intraoral scans are currently too time-consuming and costly for regular use [41]. These limitations could potentially be addressed by automated systems powered by AI, an emerging technology that holds promise for improving the efficiency and reliability of ETW assessment [41].

AI-assisted intra oral scanners in ETW

AI aids clinicians in visualizing tooth wear in ETW patients, allowing them to make quantifiable and standardized decisions related to the treatment needs for worn-out teeth. However, studies on the use of AI systems in ETW are currently limited [41]. Research indicates that in more than 95% of cases, AI systems for tooth segmentation in intraoral scanners generate clinically satisfactory outcomes with accuracy comparable to that of clinicians [41,42]. van Nistelrooij et al. (2024) demonstrated that an AI-assisted automated method for full-arch intraoral scanning was significantly faster than the manual protocol (<2 minutes vs. approximately 2 hours) without compromising clinical accuracy [41]. Likewise, Pang et al. (2024) developed an AI-based diagnostic system capable of automatically determining the degree of tooth wear using intraoral photographs, reporting superior accuracy and efficiency [43]. The AI system reduced the time required to grade an individual tooth surface to 0.07 seconds, compared to the 2.67 seconds required by clinicians [43]. Deng et al. (2024) also found the AI-based system to be a convenient clinical tool for grading teeth into categories of no, mild, and severe wear. However, the study emphasized the need for further research, including better-defined categorical annotation and additional clinical validation, to improve the accuracy of these systems [44]. Other AI applications in dentistry utilizing intraoral scanners include automated virtual implant placement, detection of caries using fluorescence or near-infrared imaging, and the computer-aided design of partial dental crowns [42]. Integrating AI systems into routine clinical practice has the potential to streamline ETW diagnosis and facilitate shared decision-making regarding necessary restorative interventions [41].

Role of teledentistry in ETW

Teledentistry offers an effective alternative to traditional in-person interactions between dentists and patients by facilitating online consultations and treatment planning [45]. These online consultations save both money and time while offering access to more affordable treatment options for both patients and clinicians [45]. Recent studies have shown that teledentistry allows remote screening and consultation for individuals with special needs, who may face physical limitations that hinder access to dental care, as well as for those at higher risk of developing ETW, such as competitive swimmers [46,47]. Further, a systematic review showed that caries detection and diagnosis of ETW using teledentistry are comparable to traditional non-teledentistry approaches [48]. Another systematic review demonstrated the positive impact of teledentistry programs on diagnosis and/or treatment planning of different oral health conditions, including tooth wear, particularly in rural populations [49].

Teledentistry can serve as a central tool for early detection of ETW, enabling clinicians to identify lesions before patients experience symptoms or visit a dental clinic. This early intervention allows professionals to manage ETW in its initial stages, preventing associated symptoms and further progression. By improving access to dental care, teledentistry has the potential to enhance oral health outcomes significantly. However, future research is needed to evaluate its impact on oral health equity [48,50].

Economic burden of underdiagnosed ETW associated with GERD

ETW, often linked to GERD, can result in substantial dental treatment costs if underdiagnosed and unmanaged. These expenses include restorative procedures, preventive care, and specialist consultations. Without early intervention, ETW can progress, becoming more widespread and involving multiple sites, which require complex rehabilitation, frequent clinical visits, and increased costs [51]. For example, the loss of healthy tooth structure compromises the longevity of the restorations, leading to more costly and invasive treatments [52]. Managing tooth wear in dental clinics within a National Health Service (NHS) hospital setting can cost between $5k and $40k per patient, excluding maintenance expenses, and may require 8-48 clinical visits, depending on the severity [51].

For GERD, the disease-related annual mean total healthcare cost in the US can be around $7k, $9k for nondysplastic Barrett’s esophagus (BE), $10k for BE-related neoplasia (including indefinite for dysplasia), $12k for low-grade dysplasia, $24k for high-grade dysplasia, and $14k for esophageal adenocarcinoma. As the disease progresses from BE to esophageal adenocarcinoma, the disease-related resource utilization increases substantially, raising costs by 16 times [53].

The costs and treatment schedules for ETW and GERD may vary across countries influenced by healthcare funding arrangements. However, these figures imply that complex interventions are not universally affordable. Therefore, early detection, prevention, monitoring, and management are essential to reduce the economic burden on patients.

Considerations for prevention and management of GERD-associated ETW

**Figure 5:**
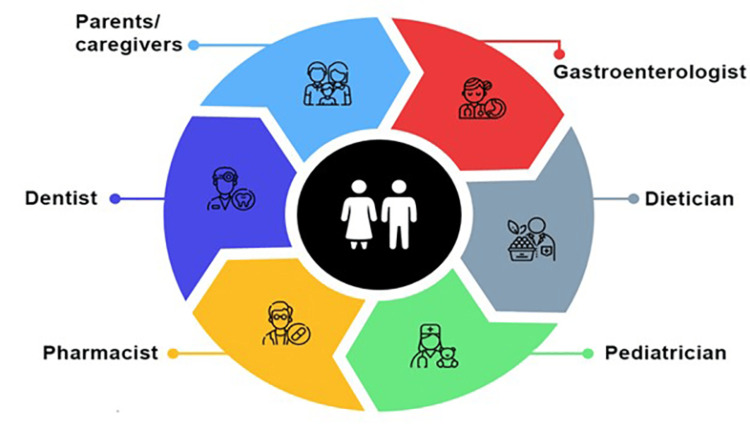
Multidisciplinary approach for prevention and management of ETW in GERD patients Adapted and recreated from references [8,40,54,55] ETW: Erosive tooth wear; GERD: Gastroesophageal reflux disease

**Figure 6:**
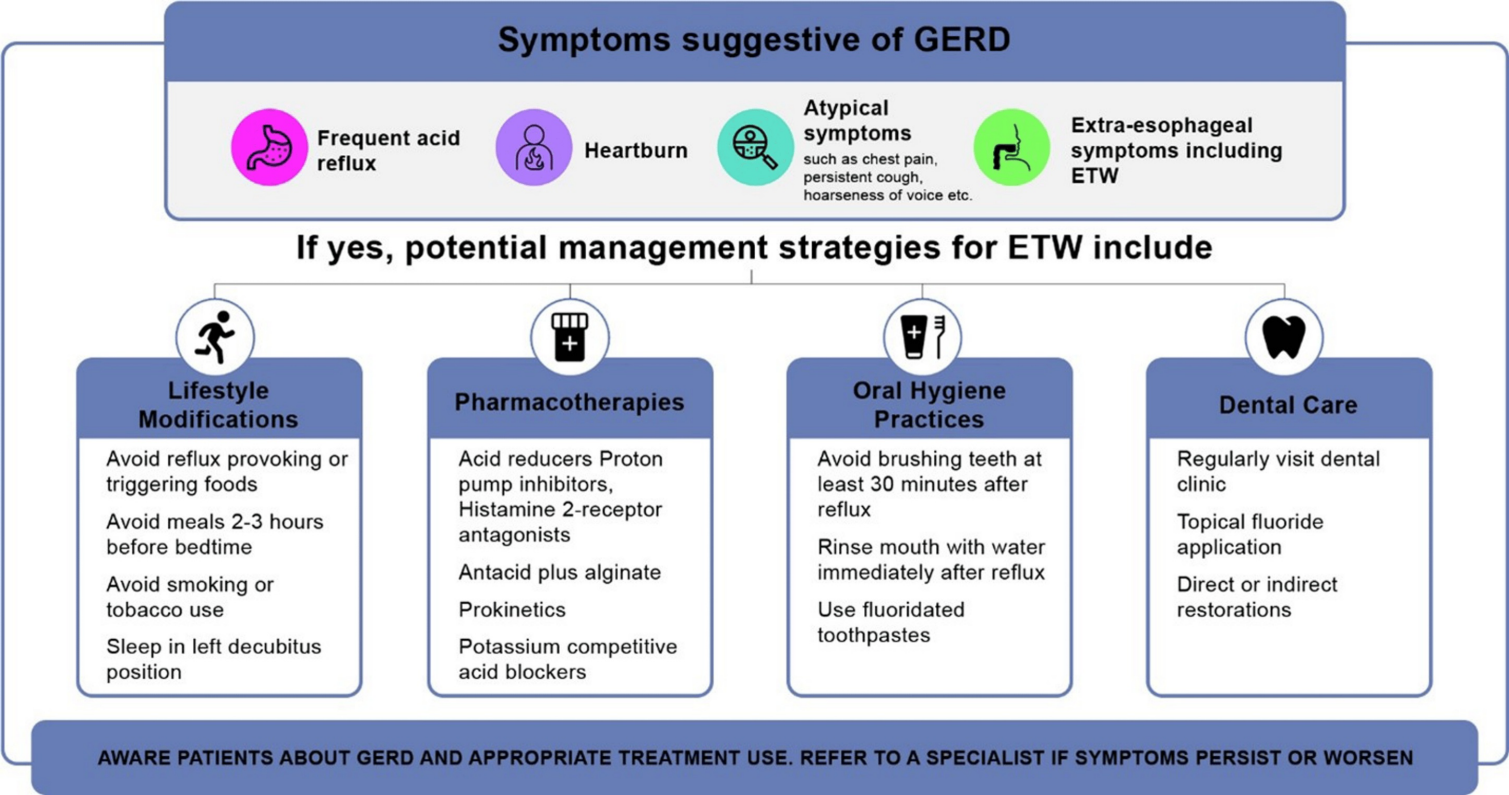
Strategies to manage ETW associated with GERD Adapted and recreated from references [54-60] ETW: Erosive tooth wear; GERD: Gastroesophageal reflux disease

Prevention and management of GERD-associated ETW at an early stage is not only essential to prevent its progression and functional impairment at later stages but also to avoid failed restorations due to wear of the surrounding tooth structure [40,54]. This can be best achieved by a multidisciplinary approach involving both medical and dental care (Figure 5) [8]. The HCPs must consider the following evidence-based practical strategies for managing ETW associated with GERD (Figure 6).

Preventive Approaches

Once identified at an early stage, preventive management for ETW includes maintaining good oral hygiene, making lifestyle or dietary changes, implementing home care routines, and professional care. Additionally, providing personalized advice based on the severity of the conditions is crucial to preventing further erosion and effectively managing ETW.

Oral Hygiene

Protecting teeth by minimizing acid exposure involves several behavioral and lifestyle modifications. Proper oral hygiene is essential, such as avoiding brushing teeth immediately after an erosive attack to prevent abrasion of softened tissue [55]. It is advisable to wait at least 30 minutes before brushing teeth after an erosive challenge [54,56]. Using low-abrasivity toothpastes and soft-bristled toothbrushes is crucial. Patients should be educated to rinse their mouth with water or a fluoride mouthwash immediately after a significant acid challenge to help neutralize the oral pH [56,57]. Additionally, antacids can be taken immediately after experiencing heartburn or a sensation of acid reflux into the oral cavity [58]. To prevent nocturnal acid challenges, patients should elevate the head of the bed or sleep on their left side (left decubitus position) [59].

Dietary Strategies to Minimize Acid Exposure

Dietary guidance is also important. Patients should avoid or limit reflux-provoking foods such as wine, citric acid, vinegar, spicy and fatty foods, tomatoes, peppermint, coffee, black tea, carbonated drinks, chocolate, and eating meals 2-3 hours before bedtime. Reducing alcohol consumption and encouraging weight loss are also important [55]. The intake of neutral beverages containing calcium, phosphate, fluoride, or water can potentially reduce erosion [60]. Consumption of acidic beverages should be limited to mealtimes, and it’s better to gulp them rather than sip slowly [57]. Using a straw can also reduce contact time between teeth and acidic beverages [54,57]. Sugarless chewing gum can increase the salivary flow rate and swallowing frequency, improving saliva's protective effect against ETW and promoting the clearance of gastric acids from the esophagus [55]. Patients presenting with ETW should have their dietary habits assessed by recording their complete dietary intake on a diet record sheet to identify the triggering foods [40]. HCPs, including dentists, should assess the erosive or reflux-provoking potential of different beverages and foods, and the frequency of their ingestion, to develop specific preventive measures and dietary interventions tailored to each patient [9]. For children, parents and caregivers must work closely with pediatricians and dietitians to implement individualized dietary modifications that ensure optimal nutrition for their growth and development.

Increasing Acid Resistance Through Fluoride Therapy

Enamel is composed of a complex hydroxyapatite crystal lattice. Fluoride from dentifrices or mouth rinse replaces hydroxide ions in hydroxyapatite crystals, forming fluorapatite, which is more acid-resistant [55,61]. This process strengthens and re-hardens weakened enamel, helping to protect teeth against acid erosion. Desensitizing agents, including toothpaste, may be recommended for dentinal hypersensitivity and pain associated with ETW. Toothpaste containing 5,000-ppm fluoride could be prescribed for ETW [54]. In office application of fluoride varnish on tooth surfaces susceptible to erosion could be considered [62]. A non-standard recommendation such as topical fluoride treatment combined with erbium and chromium laser irradiation has been shown to enhance acid resistance, improve enamel microhardness, and prevent erosion however, more research needs to be conducted to determine their effectiveness [62,63].

Protection of Tooth Structures by Application of Coating Materials

Recent in vitro studies have shown the potential effectiveness of different coating materials, including fluorides such as silver diamine fluoride (SDF), resin coatings, and newer hybrid or biomimetic coatings, in preventing ETW [64-67]. SDF, known for its bactericidal effect and ability to remineralize the affected tooth structures, has been investigated for its effect on ETW [64]. Studies have shown that topical application of SDF to be effective in preventing erosion on enamel and dentin [64,68,69].

A systematic review demonstrated the protective effect of fluoride varnishes, i.e., titanium tetrafluoride (TiF4) and sodium fluoride (NaF), on erosive lesions when applied as varnish on enamel or dentin surfaces [70]. Resin coatings, like flowable composites or sealants, can form a protective barrier to prevent further wear [65]. Newer hybrid coatings, formulated with NaF and stannous (SnCl2) ions, either directly or encapsulated in nano-containers, offer increased mechanical resistance and can protect tooth surfaces from ETW [66]. Another hydroxyapatite-based coating closely resembles the mineral structure of natural enamel and offers a superior protection against ETW due to its potential for regaining lost mineral content and better penetration into the erosive lesions in enamel and dentin [67]. While the studies have demonstrated the promising results of these coating materials, further clinical trials are needed to fully understand their long-term effectiveness in preventing and managing ETW in different clinical scenarios.

Restorative and Other Treatment Approaches

The primary focus of restorative treatment is to preserve the remaining dentition as much as possible while restoring tooth structure, function, and esthetics by repairing the damaged teeth [71,72]. It is noteworthy that there is no standard protocol for the treatment of ETW [73]. The restorative treatment should therefore be tailored to the extent and the severity of ETW. For primary dentition, the goal of treating ETW is to maintain the teeth until natural exfoliation [72]. The restoration should therefore provide pulpal protection to maintain vitality and minimize sensitivity [72]. Thus, preventive measures with lifestyle and dietary modifications are generally preferred for primary dentition. However, in children and adolescents who fail to comply with preventive measures, restorative intervention may include composite resin restorations for anterior enamel loss or stainless-steel crowns for posterior primary molars [72]. For permanent teeth, function and esthetics should be restored, and vitality is maintained. In cases of minimal tooth wear, application of resin sealant or bonding agents can be considered for reducing ETW progression [10]. Studies have shown that resin-based bonding agents can protect against tooth wear for up to three months, while fissure sealant applied to the palatal dentine surfaces of anterior teeth may prevent wear for up to nine months [74].

In moderate to severe ETW cases, restorative therapies may include direct composite restorations, indirect composites, or ceramic restorations [40,55,75]. The resin composite restorations offer a conservative, cost-effective option with good esthetic results, though long-term outcomes are uncertain [73]. Moreover, these materials are generally contraindicated for posterior teeth due to their brittle nature and the high loading forces in this region [73]. For extensive ETW in both anterior and posterior teeth, a range of restorative materials like ceramo-metal crowns, full gold crowns, lithium disilicate ceramic, zirconia, polymer infiltrated ceramic networks, resin composite, etc., can be considered [73]. In clinical studies, ceramo-metal crowns in tooth wear have demonstrated a performance comparable to the metal-free systems [76].

In advanced ETW cases, treatments may involve crowns, bridges, or prosthodontic rehabilitation to correct occlusal issues [9]. In cases with loss of vertical dimension due to ETW, treatment procedures including diagnostic wax-up, occlusal repositioning, vertical dimension restoration, and follow-up care may be considered [73]. Overall, a systematic review of clinical studies with up to 10 years of follow-up indicates that no specific restorative treatment demonstrates superior clinical performance in managing ETW [73].

Collaborative and interdisciplinary care

Multidisciplinary attention is essential for detecting and managing oral cavity acidity caused by GERD to prevent tooth wear (Figure 5) [60]. If a patient is suspected of having silent GERD, they should be referred to a gastroenterologist for a definitive diagnosis and treatment [60]. Once GERD is effectively managed, the teeth are no longer at risk. Proton pump inhibitors, like omeprazole and esomeprazole, remain the first-line treatment for GERD. Histamine-2-receptor antagonists are helpful in controlling nocturnal acid reflux. Postprandial esophageal acid can be effectively reduced using antacids or alginate combinations, some of which are available over the counter. Other therapeutic options include reflux inhibitors, prokinetics (e.g., metoclopramide, domperidone), and potassium-competitive acid blockers (e.g., vonaprazan) [59]. For patients with bruxism or clenching, especially during periods of heightened erosive risk, such as during sleep, close-fitting occlusal guards may be recommended [21]. In cases of sleep bruxism, referral to a neurologist for a sleep study may provide additional insights for tailored management [60].

Discussion

Early detection of ETW associated with GERD can be challenging, as underdiagnosed ETW associated with untreated GERD may progress further while remaining asymptomatic in its initial phase of demineralization, leading to functional and esthetic concerns in the later stage [30]. Therefore, it is essential to screen all patients in clinical practice for ETW. For adequate diagnosis, it is important to perform a comprehensive clinical examination with proper medical assessment for identification of any existing risk factors [21]. Also, it is crucial to assess the extent and severity of erosive lesions and monitor them using established clinical indices [30,40]. The integration of technology provides additional opportunities for early detection and intervention of ETW associated with GERD. For instance, teledentistry consultations can save time and enable preliminary screening for ETW [45]. The adoption of digital dentistry and AI-based systems in clinical practice may further aid in the effective monitoring and management of ETW in GERD patients [41].

Management of ETW associated with untreated GERD must adopt a holistic approach with integration of preventive and restorative measures along with interdisciplinary patient care [5]. Early management is essential, as it allows clinicians to positively influence individual oral health outcomes. A comprehensive management strategy, which includes patient education, routine dental care, preventive measures, and interdisciplinary care, can help prevent the progression of ETW to advanced stages and mitigate its long-term implications [40,54]. Routine dental visits are important for preventing the progression of ETW, as they facilitate early detection and timely management. These visits also provide opportunities for clinicians to offer tailored advice to meet the specific needs of each patient. According to the Clinical Guidelines for Dental Erosion from the Royal College of Surgeons of England (2021), adults with ETW should have dental check-ups every year, while children should have them every six months, if there are no other significant oral health risk factors [40].

Preventive strategies remain the cornerstone of managing ETW associated with GERD. Educating patients about lifestyle modifications, oral hygiene practices, and dietary guidance is crucial [55,57]. The American Dental Association (ADA) advises avoiding dietary acids between meals, reducing the consumption of acidic beverages, and eliminating habits that increase the erosive impact on teeth [35,57]. Effective preventive measures, such as the use of fluoridated toothpastes or fluoride application, are beneficial for patients susceptible to ETW [57]. Interdisciplinary care with referral to a gastroenterologist for adequate diagnosis and treatment of GERD can help control the acid reflux into the oral cavity and thus prevent the progression of ETW [60]. As per Clinical Guidelines for Dental Erosion from the Royal College of England (2021), a referral to gastroenterology is recommended if symptoms interfere with daily life, GERD tests are inconclusive, ETW progresses despite dietary changes, no clear cause of erosion is found, or severe, unilateral ETW affects the buccal surface [40].

Restorative approaches are essential for addressing the erosive lesions. The restorative approaches for permanent teeth including the application of resin sealants, bonding agents or direct or indirect restorations or prosthetic rehabilitation help restore function and esthetics [9,40,57]. In pediatric cases, composite restorations for anterior and crowns (including stainless steel crowns) for posterior teeth provide functional and esthetic solutions tailored to children’s needs [29]. However, the literature presents conflicting evidence regarding the long-term success of specific interventions, emphasizing the need for individualized treatment decisions based on the extent and severity of erosive lesions [34]. The European Guidelines (2017) proposed for management of severe tooth wear recommend that the restorative treatment should be as conservative as possible, utilizing minimally invasive methods while considering a dynamic restorative treatment concept [77]. This aims to preserve the remaining natural tooth structure while ensuring the long-term health and functionality of the teeth.

In contrast to most of the existing literature on ETW or tooth wear in general, the current paper offers a comprehensive overview of facets of ETW linked to GERD, making it a convenient resource for clinicians to use in their day-to-day clinical practice. Future research should explore the benefits of emerging technologies, such as AI and teledentistry, in the context of ETW management across diverse clinical settings.

## Conclusions

Due to its gradual progression and subtle early signs, ETW presents a significant challenge for clinicians. Diagnostic tools such as indices, intra-oral images, and IOS technologies may aid dentists with early diagnosis and patient monitoring. Untreated GERD and ETW not only impact an individual’s oral and overall health but also result in significant cost burden in later stages. Therefore, it is crucial to identify both GERD and ETW at early stages. Effective management of ETW requires a multidisciplinary approach, incorporating dietary guidance, pharmacological therapies, and fluoride interventions. These strategies are essential in preventing and effectively managing ETW associated with untreated GERD early, leading to better patient outcomes.

## Figures and Tables

**Figure 7:**
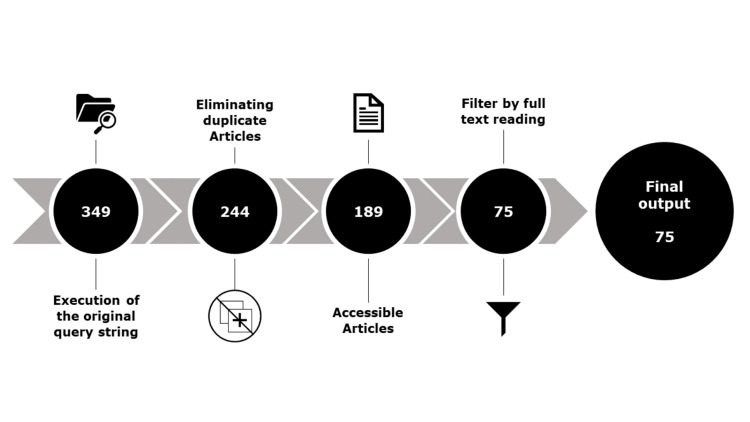
Search and selection process of articles
